# Mechanotransductive receptor *Piezo1* as a promising target in the treatment of fibrosis diseases

**DOI:** 10.3389/fmolb.2023.1270979

**Published:** 2023-10-12

**Authors:** Yi Xu, Yiqian Huang, Xiaoqing Cheng, Bin Hu, Danling Jiang, Lidong Wu, Shengliang Peng, Jialing Hu

**Affiliations:** ^1^ Department of Emergency Medicine, The Second Affiliated Hospital of Nanchang University, Nanchang, China; ^2^ The Second Affiliated Hospital of Nanchang University, The Second Clinical Medical College of Nanchang University, Nanchang, China; ^3^ Department of Ultrasound Medicine, The Second Affiliated Hospital of Nanchang University, Nanchang, China; ^4^ Department of Anesthesiology, The Second Affiliated Hospital of Nanchang University, Nanchang, China

**Keywords:** *Piezo1*, *Piezo2*, therapeutic target, fibrosis, Ca2+

## Abstract

Fibrosis could happen in every organ, leading to organic malfunction and even organ failure, which poses a serious threat to global health. Early treatment of fibrosis has been reported to be the turning point, therefore, exploring potential correlates in the pathogenesis of fibrosis and how to reverse fibrosis has become a pressing issue. As a mechanism-sensitive cationic calcium channel, *Piezo1* turns on in response to changes in the lipid bilayer of the plasma membrane. *Piezo1* exerts multiple biological roles, including inhibition of inflammation, cytoskeletal stabilization, epithelial-mesenchymal transition, stromal stiffness, and immune cell mechanotransduction, interestingly enough. These processes are closely associated with the development of fibrotic diseases. Recent studies have shown that deletion or knockdown of *Piezo1* attenuates the onset of fibrosis. Therefore, in this paper we comprehensively describe the biology of this gene, focusing on its potential relevance in pulmonary fibrosis, renal fibrosis, pancreatic fibrosis, and cardiac fibrosis diseases, except for the role of drugs (agonists), increased intracellular calcium and mechanical stress using this gene in alleviating fibrosis.

## 1 Introduction

### 1.1 Fibrosis

The intricate balance between tissue repair and remodeling is disrupted in fibrosis, a pathological condition characterized by the aberrant accumulation of fibrous connective tissue within organs or tissues. This process, driven by a cascade of molecular events triggered by injury, inflammation, or underlying diseases, culminates in the excessive deposition of collagen and an altered extracellular matrix (ECM) composition (Wen D. et al., 2022). The lungs, liver, kidneys, and heart are among the organs susceptible to fibrotic transformations, with persistent injury perpetuating a cycle of escalating fibrogenesis, ultimately leading to compromised organ function and a continuum of deleterious consequences.

In the physiological processes of an organism, to maintain the normal functioning of tissues and organs, it is mandatory to ensure an appropriate reparative response, and fibrosis is considered to be a reparative response that restores the organ structure by replacing the destroyed tissues (Henderson et al., 2020; Wen J. H. et al., 2022). However, if this repair response is uncontrolled or over-activated, it can lead to pathological states such as organ fibrosis and abnormal function (Wen J. H. et al., 2022). Therefore, fibrosis is a pathological condition characterized by parenchymal cell necrosis as well as an unusual amount of hyperplasia and hyper-deposition of the extracellular matrix (Antar et al., 2023).

Fibrosis can develop in multiple organs and often occurs in the end stages of the disease. In the lung, fibrotic diseases include pneumoconiosis (Qi et al., 2021) and silicosis (Zhao Y. et al., 2022), whose etiology is known, and idiopathic pulmonary fibrosis (Cottin et al., 2019; Somogyi et al., 2019), whose etiology is not yet known. Pulmonary fibrosis is commonly the end stage of chronic lung diseases, such as silicosis (Handra et al., 2023) and idiopathic pulmonary fibrosis (Heukels et al., 2019) mentioned above. In chronic lung diseases, lung tissue will be progressively replaced by scar tissue, causing difficulty in breathing, and may eventually cause respiratory failure. Chronic liver diseases, such as chronic hepatitis B (Stalla et al., 2022), hepatitis C (Sebastiani et al., 2014), and alcoholic liver disease (Lackner and Tiniakos, 2019), are often accompanied by liver fibrosis at the end stage of the disease, eventually leading to severe damage to liver function and symptoms such as jaundice and hepatic ascites (Mansour and McPherson, 2018). Cardiac fibrosis is often the end stage of heart failure, prolonged myocardial damage can lead to fibrosis of myocardial tissue (González et al., 2018; Bacmeister et al., 2019). Similarly, chronic kidney disease is one of the common causes of renal fibrosis, prolonged damage to nephrons and glomeruli will gradually lead to fibrosis of the kidneys (Rayego-Mateos and Valdivielso, 2020; Panizo et al., 2021). Pancreatic fibrosis is a disease closely related to chronic pancreatitis. In patients with chronic pancreatitis, pancreatic tissue is gradually damaged, and pancreatic fibrosis is a manifestation of advanced pancreatitis (Shimizu, 2008; Swain et al., 2022).

In addition to the above-mentioned organs, fibrosis also often occurs in the skin (Andrews et al., 2016), bones and muscles (Mahdy, 2019), gastrointestinal tract (Wang J. et al., 2021), and other organs. In this review, we are focusing on *Piezo1* and its potential contribution to the pathophysiology of pulmonary fibrosis, renal fibrosis, pancreatic fibrosis, and cardiac fibrosis diseases.

### 1.2 Introduction of *Piezo1*

Using stress-sensitive cells, Prof. Ardem Patapoutian uncovered a new sensor that is capable of responding to mechanical irritation in the skin and visceral organs (Dubin and Patapoutian, 2010; Kefauver et al., 2020). Thus, a new and completely unknown mechanosensitive ion channel, *Piezo1*, was discovered, followed by a second related gene, *Piezo2* (Coste et al., 2010). *Piezo* proteins are a combination of *Piezo1* and *Piezo2*. *Piezo1* is a mechanosensitive cation channel protein situated on the membrane of cells and is a pivotal cytomechanical sensor that converts mechanical stimulation into galvanic signaling (Coste et al., 2010).

*Piezo1* is a protein that can be engaged in the process of mechanosensation and mechanical force transformation. It forms ion channels on the cell surface and can perceive and react to mechanical stimulation around the cell (Huang et al., 2023). As mentioned above, *Piezo1* channels perform an essential function in several physiological processes, including cell migration (Holt et al., 2021; Yu et al., 2021), vascular smooth muscle cell contraction (Chen et al., 2022a; Chen et al., 2022b; Porto Ribeiro et al., 2022), red blood cell morphology changes (Cahalan et al., 2015; Svetina et al., 2019), and sensory neuron perception of touch and pressure (Coste et al., 2010). In addition to the perception of mechanical stimuli, *Piezo1* is engaged in the modulation of a wide range of cellular functions. For example, it regulates stem cell fate determination (Sugimoto et al., 2017; Qiu et al., 2023), cell proliferation and differentiation (He et al., 2018), skeletal muscle development and repair (Bernareggi et al., 2022), vascular endothelial cell permeability (Friedrich et al., 2019), and tumor cell invasion and metastasis (Jiang et al., 2022). It has also been found that *Piezo1* mutations are also associated with several diseases, such as congenital erythrocytosis (Knight et al., 2019; Filser et al., 2021; Sochorcova et al., 2023) and familial pulmonary hypertension (Wang Z. et al., 2021; Liao et al., 2021; Porto Ribeiro et al., 2022). Currently, there are also a large number of studies that have identified a potential relationship between *Piezo1* and fibrotic diseases (Zhang et al., 2021a; Braidotti et al., 2022a; He et al., 2022a; Swain et al., 2022).

In conclusion, *Piezo1* is an important protein that has a critical role in mechanical force perception and regulation of cellular functions. Further studies are needed to gain insight into its specific role and regulatory mechanisms in physiological and pathological processes, which will not only facilitate our understanding of the mechanosensory mechanisms of *Piezo1* but more importantly, can provide new methodologies to develop treatments for associated disorders.

### 1.3 *Piezo1* and fibrosis

We all know that the main pathological changes in fibrosis are increased synthesis and insufficient degradation of extracellular matrix and that persistent fibrosis leads to structural destruction and functional decay of organs, but the mechanisms behind many fibrotic diseases are not yet understood by us. Several experiments now suggest that *Piezo1* may have a potential relationship with fibrosis (Zhang et al., 2021a; He et al., 2021; Bartoli et al., 2022; Braidotti et al., 2022a; Zhao X. et al., 2022; Fang et al., 2022; Swain et al., 2022; Xing et al., 2023). In the pathological state, activation of *Piezo1* channels by mechanical stimuli induces excessive ECM synthesis in the cells involved, leading to ECM deposition and promoting the progress of fibrosis. It was found that aberrant exposure of *Piezo1* can be observed in fibrotic tissues and organs.

One investigator created a genetically engineered mouse model (He et al., 2022a) that specifically knocked out *Piezo1* from bone marrow cells, intending to study the mechanism of the mechanosensitive protein *Piezo1* in renal fibrosis, and finally found that mice with *Piezo1* knockout alleviates renal fibrosis, suggesting that the development of targeting *Piezo1* mechanical channels offers a possible approach to the management of renal fibrosis (He et al., 2022a; Zhao X. et al., 2022). In the pancreas, a hypertensive condition stimulates the opening of *Piezo1* channels and the formation of fibrosis induced by stress (Swain et al., 2022). As for cardiomyocytes, experiments have identified a stress response after a myocardial injury that leads to the upregulation of *Piezo1*, which may be responsible for the positive feedback of fibrosis progression (Braidotti et al., 2022a). Experimental studies have demonstrated that *Piezo1* has an active role in ARDS-associated pulmonary fibrosis exacerbated by mechanical stretch (MV) via mediation of calcium inward flow as well as ATP emission (Fang et al., 2022). Activation of *Piezo1* channels can influence a range of signal pathways that play an important role in the progression of fibrotic disease (He et al., 2022a). For example, activation of *Piezo1* can lead to calcium inward flow, which activates signal pathways such as TGF-β/Smad and p38-MAPK, which perform key functions in the onset and progression of fibrosis (Ding et al., 2021).

## 2 Structure and characteristics of *Piezo1*

*Piezo1* and *Piezo2* constitute the 2 major mechanically-activated (MA) channels identified in mammals. The *Piezo1* protein was initially identified in mice (Coste et al., 2010). By comparison, the *Piezo1* gene was found to be homologous in humans (Schrenk-Siemens et al., 2015), mice (Ikeda et al., 2014), chickens (Soattin et al., 2016), birds (Schneider et al., 2014), *drosophila* (He et al., 2018), African clawed frog meadowlark (Methfessel C Fau - Witzemann et al., 1986), and zebrafish (Faucherre et al., 2013). *Piezo1* is broadly expressed in several human organs and tissues, encompassing vital organs such as the lungs (Xiong et al., 2022), the gastrointestinal system (Yang et al., 2022), and the skeleton (Qin et al., 2021; Xu et al., 2021), which strongly suggests that Piezo1 may have a critical function in the normal functioning of these organs, such as in respiration, digestion, and locomotion (Qin et al., 2021; Xu et al., 2021; Xiong et al., 2022; Yang et al., 2022). Structural similarities between mouse and human Piezo1 channels were observed by cryo-electron microscopy, providing a basis for further functional studies (Wang and Xiao, 2018; Xiao, 2020). *Piezo1* and *Piezo2* are respectively positioned on chromosome 16 and chromosome 18. In the human body, *Piezo1* is comprised of 2,520 amino acids and *Piezo2* is comprised of 2,752 amino acids (Gottlieb and Sachs, 2012).

The mechanosensitivity of *Piezo1* channels is explained by a lever-like mechanism of mechanical action based on a unique three-leaf propeller-like homologous structure (Bae et al., 2015; Jiang et al., 2021a). The basic structure of the Piezo1 channel consists of multiple repeating structural domains, which include an N-terminal region, a membrane domain, and a C-terminal region (Kefauver et al., 2020; Fang et al., 2021; Tang et al., 2022). Based on the structure and function of the *Piezo1* protein, some researchers have divided it into an ion-conducting pore portion, an anchor that acts as a conversion element: the CTD and bundles, and a mechanosensing portion consisting of the TM blades (Zhao et al., 2019). The channel can be in three active states: closed, open, and inactivated (Cox and Gottlieb, 2019). A mechanical stimulus acting on the cell membrane triggers the *Piezo1* channel to shift from a closed state to an open state, allowing the flow of ions, such as calcium, potassium, and sodium ions (Gottlieb and Sachs, 2012).

The interaction of *Piezo1* with the cytoskeleton in mechanosensing has been described in detail (Nourse and Pathak, 2017; Jiang et al., 2021b). The overexpression of *Piezo1* channels in cells is characterized by rapid and complete inactivation, described as a pressure pulse in a split second (Coste et al., 2010; Wu et al., 2017), this character has also emerged as a signature of the *Piezo1* channel. The structure of the *Piezo1* channel facilitates our understanding of its mechanism in sensing mechanical stimuli and regulating the permeability of ion channels.

As an important force-sensitive channel, *Piezo1* plays multiple physiological functions in cells. First, it plays a key role in maintaining the shape of red blood cells (Vaisey et al., 2022). By sensing extracellular mechanical forces, *Piezo1* can regulate the morphology of the cell membrane and ensure the adaptability and functionality of red blood cells (Vaisey et al., 2022; Evtugina et al., 2023; Hatem et al., 2023). Secondly, *Piezo1* is involved in the regulation of immune responses (Atcha et al., 2021a; Lai et al., 2022). The opening of its channels can trigger intracellular signal transmission, thereby affecting the activity of immune cells, which is crucial for maintaining the balance of the immune system (Solis et al., 2019a; Aykut et al., 2020; Atcha et al., 2021a; Geng et al., 2021; Leng et al., 2022). In addition, *Piezo1* is also involved in the functional regulation of the cardiovascular system (Li et al., 2014; Douguet et al., 2019), and its channel activity is closely related to pathological conditions such as arrhythmia (Jiang F. et al., 2021; Rolland et al., 2023), suggesting that it plays an important role in cardiovascular biology.

One of the main functions of *Piezo1* is to sense and respond to mechanical stimulation. The opening of its channel will lead to an increase in intracellular calcium ion concentration, thereby triggering multiple signaling pathways. This process not only affects the biological effects of cells, such as cell apoptosis, proliferation, and migration (Volkers et al., 2015; Liu S. et al., 2021; Dombroski et al., 2021; Shinge et al., 2022; Song et al., 2022). *Piezo1* can also activate the protein kinase pathway and further regulate the activity of multiple cell signaling pathways (Blythe et al., 2019; Liu S. et al., 2021; Chen S. et al., 2022; Wang et al., 2022). In addition, Piezo1 can also regulate the activity of Na, and K-ATPase, further affecting intracellular ion balance and cell membrane stability (Shahidullah et al., 2022; Hirata et al., 2023). Recently, Shahidullah M and his colleagues studied the relationship between *Piezo1* and Na, K-ATPase-mediated ion transport in mouse crystals. They found that after activation of *Piezo1*, Na, K-ATPase in cells will be affected (Shahidullah et al., 2022).

Therefore, *Piezo1* has a variety of key physiological functions in cells. Its research will not only help to gain a deeper understanding of the basic mechanisms of cell biology but may also provide new therapeutic targets for the treatment of related diseases. Therefore, the function and regulatory mechanism of *Piezo1* deserve further in-depth study.

## 3 A new hope for fibrosis diseases: *Piezo1*

### 3.1 *Piezo1* and pulmonary fibrosis

Pulmonary fibrosis (PF) is a diffuse interstitial pulmonary disease featuring progressive inflammation and extracellular matrix deposition, resulting in irreversible damage caused by abnormal lung tissue repair (Thannickal et al., 2004; Henderson et al., 2020; Zhao Y. et al., 2022).

Several studies have demonstrated a strong relationship between epithelial-mesenchymal transition (EMT) with fibrosis (Qian et al., 2018; Rout-Pitt et al., 2018; Salton et al., 2019; Liu et al., 2022). Transforming growth factor beta (TGF-β) is thought to be closely associated with early embryonic development and organogenesis, and adult homeostasis (Xu et al., 2018), TGF-β overexpression can lead to excessive metabolic disorders and dysfunction, promoting EMT and ECM deposition (Su et al., 2020; Lee and Massagué, 2022), leading to fibrosis and cancer development (Hao et al., 2019; Andugulapati et al., 2020; Kim et al., 2020). *Piezo1* is a mechanosensitive calcium channel, and immunohistochemical staining revealed widespread *Piezo1* expression in mouse pulmonary tissues (Zhang Y. A. et al., 2021), epithelial cells, and endothelial cells (Zhong et al., 2018; Friedrich et al., 2019; Bhattacharya and Hough, 2019), and was suggested to play an important role in bleomycin-induced pulmonary fibrosis (Solis et al., 2019a; Solis et al., 2019b).

Jia-Qi Huang and his colleagues discovered through cell line studies and cell culture of rat lung cells that a positive response mechanism for the relationship of *Piezo1* to TGF-β1 was found to exist in radiation-induced pulmonary fibrosis (Huang et al., 2021a) and has a critical role in the radiation-induced generation of EMT. It was found that upregulation of TGF-β1 was associated with the activation of *Piezo1*, some researchers have found through cell line studies (Lei et al., 2019; Huang Y. et al., 2021) and animal studies (Lei et al., 2019) that the Ca2+/HIF-1α signaling pathway can activate TGF-β1, and *Piezo1* induced EMT by regulating TGF-β1 through the Ca2+/HIF-1α signaling pathway (Lei et al., 2019; Huang et al., 2021a; Huang Y. et al., 2021). TGF-β1 was able to inhibit C/EBPβ expression (Ramji and Foka, 2002), and C/EBPβ acts on the *Piezo1* promoter to reduce the expression of *Piezo1* (Huang et al., 2021a; Ghafouri-Fard et al., 2021). Research has also revealed that TGF-β acts through the *smad3* signaling pathway to inhibit C/EBPβ on the expression of the *Piezo1* promoter, resulting in upregulation of *Piezo1* expression (Huang et al., 2021c).

Mechanical ventilation is essential in the treatment of some critical patients with respiratory illnesses, including acute respiratory distress syndrome (ARDS) (Walter et al., 2018; Pelosi et al., 2021; Shi et al., 2023). As mentioned previously, *Piezo1* is strongly observed in both normal pulmonary epithelial cells and pulmonary endothelial cells (Bhattacharya and Hough, 2019; Shi et al., 2023). Classification of alveolar epithelial cells into type I (AT I) and type II(AT II). Caveolae are expressed in type I alveolar epithelium (Wicher et al., 2019; Jones and Minshall, 2020), and caveolae were found to be mechanosensory in the alveoli (Thompson et al., 2014; Wicher et al., 2019), stretch-induced Ca2+ signaling is dependent on Ca2+ entry through *Piezo1* channels, allowing AT I cells to release ATP, resulting in the regulation of surfactant secretion in AT II cells (Diem et al., 2020; Lin et al., 2022).

Some researchers have found through animal trials (Zhang et al., 2021c) and cell line trials (Diem et al., 2020; He J. et al., 2022) that mechanical stretch can significantly induce *Piezo1* activation in epithelial cells (Diem et al., 2020; Zhang et al., 2021c; He J. et al., 2022). *Piezo1* can induce ATP release during the mechanical stretch, and the released ATP can, in turn, drive mechanical stretch to enhance EMT, thus exacerbating pulmonary fibrosis (Diem et al., 2020; Fang et al., 2022), and leading to more severe pulmonary fibrosis in ARDS during ventilation. Although *Piezo1*-mediated ATP release is essential in the exacerbation of pulmonary fibrosis by mechanical stretch (Miyamoto et al., 2014; Diem et al., 2020), the association of ATP with EMT and pulmonary fibrosis remains to be investigated.

When mechanical stretching was performed on pulmonary epithelial and endothelial cells, the extent of the injury was directly related to the duration of mechanical stretching, and the expression of *Piezo1* was also proportional to it, indicating an association between *Piezo1* and respiratory lung injury (Zhang Y. A. et al., 2021). After excessive mechanical stretching of the lung endothelium, Ca2+ inward flow activates *Piezo1* channels and the adhesion junctions between endothelial cells are disrupted (Friedrich et al., 2019; Zhong et al., 2020; Jiang et al., 2021b). Using a mouse model induced by hyper-tidal volume mechanical ventilation (Zhang et al., 2021c), Yang Zhang and members of his experiments demonstrated that *Piezo1* functions in the pathological processes in the epithelial cells of the lung in ventilator-induced pulmonary damage by activating the RhoA/ROCK1 pathway (Zhang Y. A. et al., 2021). In conclusion, *Piezo1* performs a crucial function in lung injury due to mechanical stretch (MV).

**FIGURE 1:**
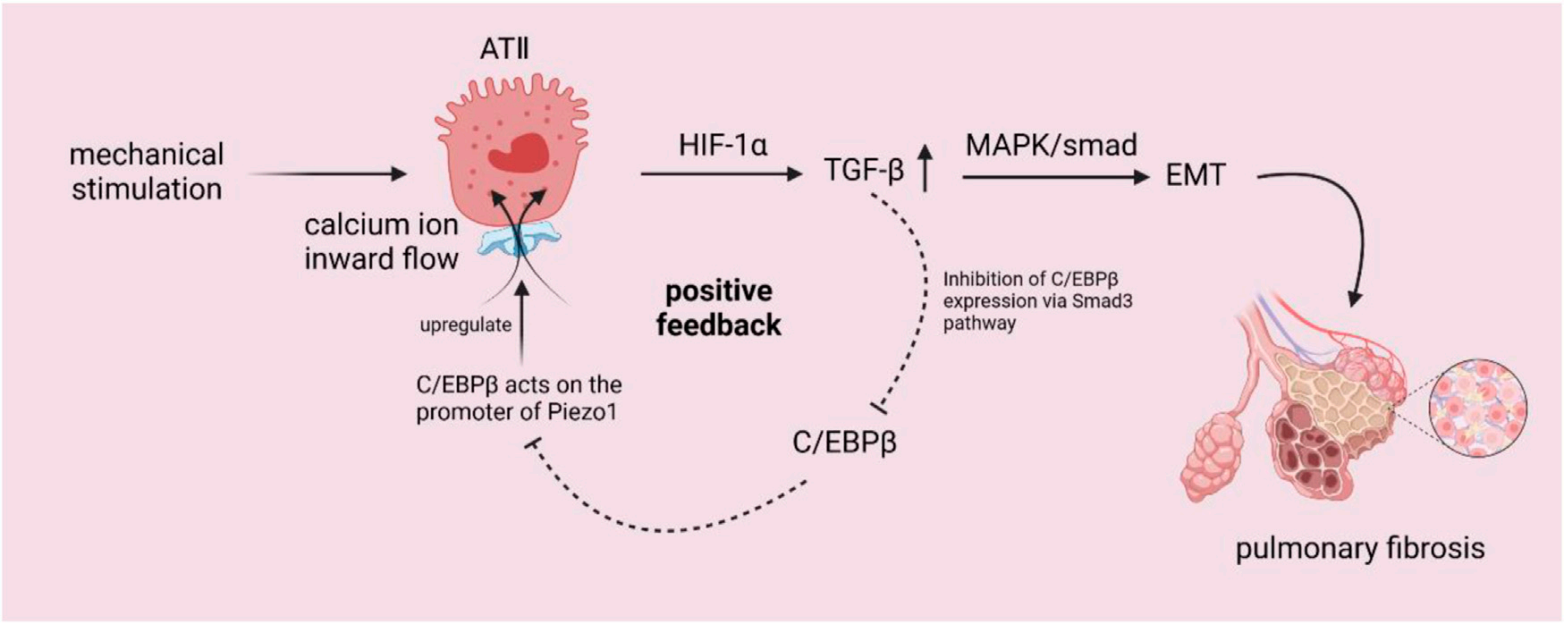
Schematic diagram of the mechanism by which mechanical stimulation of ATII cells activates *Piezo1* channels to trigger related pathways. After activation of *Piezo1* by mechanical signals, calcium ions inwardly flowed into the cells, and *Piezo1* regulated TGF-β1 through the Ca2+/HIF-1α signaling pathway, leading to upregulation of TGF-β1. The upregulation of TGF-β1 could on the one hand activate the EMT through the MAPK and *smad*-dependent signaling pathways to promote lung fibrosis, on the other hand, and might inhibit C/EBPβ by the Smad3 pathway, which could inhibit the effect of C/EBPβ on the promoter of *Piezo1*, and result in the upregulation of *Piezo1*’s expression as well.

When understanding the current relationship between *Piezo1* and pulmonary fibrosis, we can find that in ATⅠ, ATⅠ-expressed caveolae can respond to mechanical signals through plasma membrane invagination, caveolae act as a mechanical sensor of *Piezo1*, Ca2+ inward flow activates pannexin-1 hemichannel to enter and localize to caveolae, acting on ATⅠ to release ATP (Diem et al., 2020); as shown in the Figure 1, among ATⅡ cells, after the mechanical signal activates *Piezo1*, calcium ion inward flow enters the cell, *Piezo1* regulates TGF-β1 expression through Ca2+/HIF-1α signaling pathway, so that TGF-β1 expression is upregulated (Huang et al., 2021c; Zhang et al., 2022), and the upregulation of TGF-β1 can be activated through MAPK and *smad*-dependent signaling pathway (Guo et al., 2021) on the one hand EMT, which promotes lung fibrosis, and on the other hand, it may suppress the expression of C/EBPβ by the Smad3 pathway (Feinberg et al., 2004; Lourenço et al., 2020), thus inhibiting C/EBPβ from acting on the *Piezo1* promoter and causing *Piezo1* to be upregulated as well (Huang et al., 2021c).

In addition, in animal trials on lung injury caused by mechanical ventilation in rats (Zhang et al., 2021c), mechanical ventilation can also stimulate *Piezo1* channel activation, convert mechanical signals into biological signals, calcium ion inward flow, and elevated calcium in alveolar epithelial cells, leading to downregulation of non-apoptotic cytokine Bcl-2 expression (Liang et al., 2019) and alveolar cell necrosis. *Piezo1* is also an upstream modulator of the RhoA/Rock1 pathway, activating this signaling pathway and inducing the onset of pulmonary fibrosis (Zhang et al., 2021c). In contrast, in respiratory lung injury secondary to ARDS, Piezo in the lung endothelium is activated by mechanical signaling and calcium ions flow inward, leading to disruption of the adhesion junctions (AJs) between endothelial cells and resulting in damage to the lung endothelial barrier (Liang et al., 2019; Jiang et al., 2021c).

*Piezo1* is an ion channel protein widely expressed in various tissues and cell types, and its role in various disease processes has attracted much attention. *Piezo1* is widely expressed in lung tissue, epithelial cells, and endothelial cells, and interacts with the TGF-β1 signaling pathway. In pulmonary fibrosis, the upregulation of *Piezo1* is an important event, which may serve as a response to mechanical stimuli and play a key role in the occurrence and progression of fibrosis. Mechanical stretch activates *Piezo1*, leading to Ca2+ influx, activating the Ca2+/HIF-1α signaling pathway of TGF-β1, inducing epithelial-mesenchymal transition (EMT), and promoting pulmonary fibrosis. TGF-β1 also inhibits C/EBPβ through the smad3 signaling pathway, thereby upregulating the expression of *Piezo1*. Therefore, the increase in *Piezo1* is accompanied by pulmonary fibrosis and further promotes the occurrence of fibrosis.

### 3.2 *Piezo1* and renal fibrosis

Renal fibrosis is an irreversible pathology of long-term kidney disease and end-stage renal disease, manifested by improved production and insufficient breakdown of ECM within the renal tubules (Black et al., 2019; Bülow and Boor, 2019; Liang et al., 2022). *Piezo1* which is a mechanosensitive cation channel (Coste et al., 2010) senses the stiffness from the external environment and converts mechanical signals into intracellular electrochemical signals (Lewis and Grandl, 2015; Kefauver et al., 2020; Xu et al., 2021). *Piezo1* is expressed in endothelial and mural cells, proximal and distal curvilinear tubules of the renal vesicle (Peyronnet et al., 2013; Martins et al., 2016; Dalghi et al., 2019). Increased ECM synthesis and sclerosis of the cellular environment may exacerbate renal fibrosis (Chen et al., 2014; Imamura et al., 2018). One study using an animal model found that increased ECM synthesis and sclerosis can activate *Piezo1* and exacerbate kidney fibrosis by the *Piezo1*-p38MAPK-YAP signaling pathway (Fu et al., 2021).

Macrophages have an essential function in renal fibrosis, and macrophages can transmit information to cells by sensing mechanical signals (Wei et al., 2022; Wu et al., 2022). It has been suggested that macrophages are multifunctional cells that possess pro- and anti-fibrotic effects (Wynn and Vannella, 2016; Tang et al., 2019). In the unilateral ureteral obstruction (UUO) model (Lee et al., 2023), *Piezo1* deletion was observed followed by a crucial reduction in the *CCL2-CCR2* signaling pathway and *Notch* pathway (He et al., 2022a), which inhibited the inflammation of macrophages and the progression of renal fibrosis. Macrophages are classified into M1 type (pro-fibrotic) and M2 type (anti-fibrotic) (Nishida et al., 2005). *Piezo1* can activate the *CCL2-CCR2* pathway via *Notch*, causing macrophage aggregation to trigger inflammation and thereby mediating ECM deposition and renal fibrosis (He et al., 2022a).

Recent research revealed that *Piezo1* expressed markedly elevated in fibrotic kidneys, and treatment of the UUO model with GsMTx4, a blocker of *Piezo1* (Velasco-Estevez et al., 2020), revealed a significant attenuation of renal fibrosis, indicating that *Piezo1* has an essential function in renal fibrosis (Zhao X. et al., 2022). In addition, it has been found that mechanical stretch stimulation of *Piezo1* induced fibrosis in human renal cortical proximal tubular epithelial cells (HK2 cells) (Zhang et al., 2021; Zhao X. et al., 2022) and primary cultured mouse proximal tubule cells (mptc) (Zhao X. et al., 2022), while inhibition of *Piezo1* inhibited fibrosis through blocking the TGF-β1 signaling pathway, which suggests the role of *Piezo1* in the fibrosis of renal tubular epithelial cells caused by mechanical stretch.

As is known, TGF-β1 is an important marker of EMT, but several studies have found no strong correlation between EMT and renal fibrosis *in vivo* (Galichon et al., 2013; Sheng and Zhuang, 2020). It has been suggested that TGF-β1 damages renal tubules through the smad signaling pathway, resulting in inadequate deposition and degradation of ECM, leading to renal fibrosis (Hu et al., 2018; Gifford et al., 2021).

**FIGURE 2:**
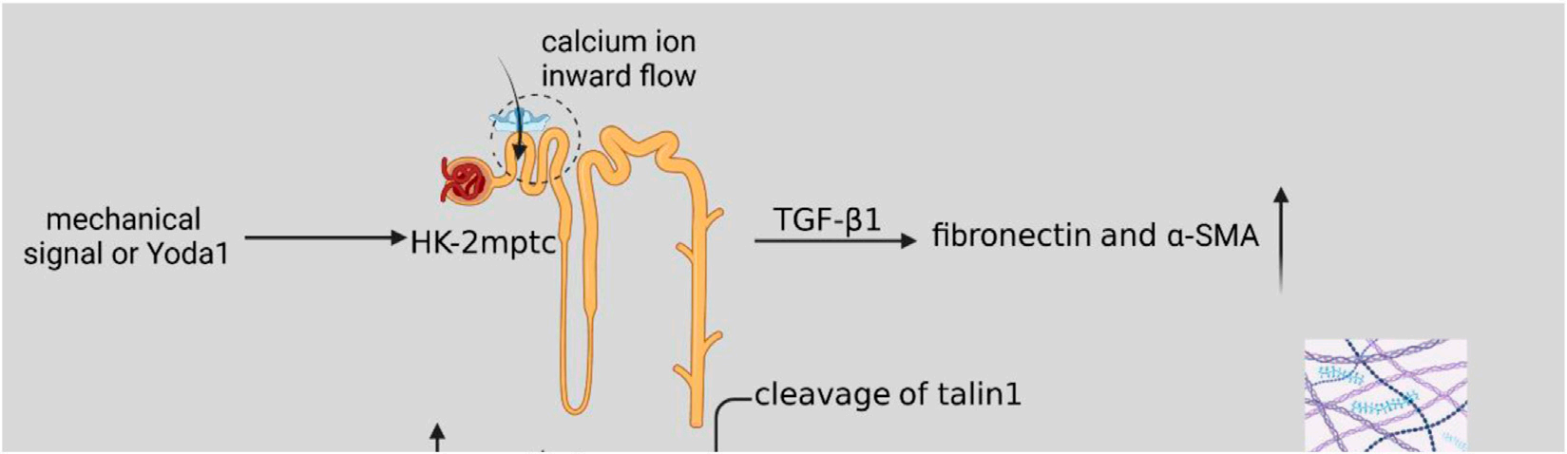
Schematic diagram of the *Piezo1*-related pathway mechanisms in renal fibrosis. Mechanical signaling or *Yoda1* activates cellular *Piezo1* channels after acting on HK2 cells and mptc. *TGF-β1* induces upregulation of fibronectin and *α-SMA*, resulting in increased ECM synthesis. Activation of calpain2, which signals downstream of *Piezo1*, induces talin1 clearance and upper-regulation of integrin β1 protein, and increased ECM stiffness. A large number of calcium ions inward flow may activate P38-MAPK molecules, P38-MAPK then activates YAP, and YAP induces ECM deposition, which promotes the process of renal fibrosis.

As shown in Figure 2, the mechanical signal or activator of *Piezo1*, Yoda1, acted on HK2 cells and mptc, activated cellular piezo1 channels, TGF-β1 induced upregulation of fibronectin and α-SMA (Zhao X. et al., 2022), which increased ECM synthesis and also inhibited ECM degradation. The mechanical signal was delivered to ECM with calcium inward flow, activation of calpain2, which signals downstream of *Piezo1*, induces talin1 clearance and upper-regulation of integrin β1 protein (Bate et al., 2012; Zhao X. et al., 2022), and integrin and ECM bind more tightly and induce the development of renal fibrosis. When ECM stiffness increases, it may activate Yes-associated protein (YAP) (Dupont et al., 2011; Calvo et al., 2013), which acts as a transcription factor of the Hippo signaling pathway mechanically regulated by ECM stiffness. When *Piezo1* is activated, a large amount of calcium ions inward flow may activate the P38-MAPK molecule, and P38-MAPK reactivates YAP, and YAP induces ECM deposition and promotes the process of renal fibrosis (Fu et al., 2021).

*Piezo1* is expressed in renal tubular and renal capsule endothelial cells and parietal cells. Upregulation of *Piezo1* in renal fibrosis also occurs during fibrosis. Mechanical stretch or *Piezo1* activators can lead to an increase in intracellular calcium ions, increased expression of TGF-β1, and promote ECM synthesis and renal fibrosis. It is worth mentioning that although TGF-β1 is an important marker of EMT, some studies have not found a strong correlation between EMT and renal fibrosis *in vivo*, indicating that *Piezo1* may have a more complex role in renal fibrosis.

### 3.3 *Piezo1* and pancreatic fibrosis

The pancreas is sensitive to mechanical injury (Romac et al., 2018), and pressure on the gland may lead to the development of pancreatitis (Wang et al., 2009; Romac et al., 2018; Swain et al., 2020; Swain et al., 2020) and fibrosis (Swain et al., 2022), so the pancreas can sense mechanical tension. When the pancreas is subjected to external mechanical injury, *Piezo1* pathologically opens continuously, calcium ions flow in a large amount, intracellular calcium ion homeostasis is disrupted, and intracellular zymogen and lysosomal particles in the pancreatic follicle cells react abnormally, and lead to pancreatitis (Geokas et al., 1985; Tenner et al., 2013; Mayerle et al., 2019). Pancreatic fibrosis increases the risk of pancreatic cancer, and studies in recent years show that the progression from pancreatitis to pancreatic cancer may be interspersed with pancreatic fibrosis (Cannon et al., 2021; Huang et al., 2021). Excessive deposition of ECM produced primarily by activated pancreatic stellate cells (PSCs) triggers pancreatic fibrosis (Phillips et al., 2012; Thomas and Radhakrishnan, 2019; Huang et al., 2021; Hamada et al., 2022; Swain et al., 2022). PSCs can express *Piezo1* (Kuntze et al., 2020; Swain et al., 2022), intracellular calcium ion concentration increases and TGF-β1 expression increases after a mechanical pull or the *Piezo1* activator yoda1 acts on PSCs, and these phenomena disappear when the *Piezo1* inhibitor GsMTx4 acts, so *Piezo1* is critical in stress-induced pancreatic fibrosis (Swain et al., 2022).

However, one study found that *Piezo1* is a rapidly inactivating pathway (Del Mármol et al., 2018; Shi et al., 2020) and that *Piezo1* only causes a transient elevation of intracellular calcium ions (Swain et al., 2020), therefore, it is presumed that other mechanisms could lead to a sustained increase in intracellular calcium ions. TRPV4 was also found to be expressed in both mouse and human pancreatic follicles (Swain et al., 2020), and in the absence of TRPV4, *Piezo1* triggers insufficient calcium inward flow signaling (Swain et al., 2020; Gorelick and Nathanson, 2020; Swain et al., 2022). It has been suggested that *Piezo1* stimulates PLA2, which initiates the TRPV4 pathway (Swain et al., 2020), leading to a sustained increase in intracellular calcium ions, a sustained increase in intracellular calcium ion concentration will further activate intracellular protein kinases, leading to cellular self-digestion and damage to pancreatic cells (e.g., fibrosis). In addition to this, in human and mouse models, macrophages exacerbate fibrosis (Hu et al., 2020; LaRue et al., 2022) by producing TNF-α and TGF-β1 (Xue et al., 2015), while in macrophages, the mechanical pull is engaged in the inflammatory response and fibrosis by acting on *Piezo1* (Solis et al., 2019a; Atcha et al., 2021b; Atcha et al., 2021a).

**FIGURE 3:**
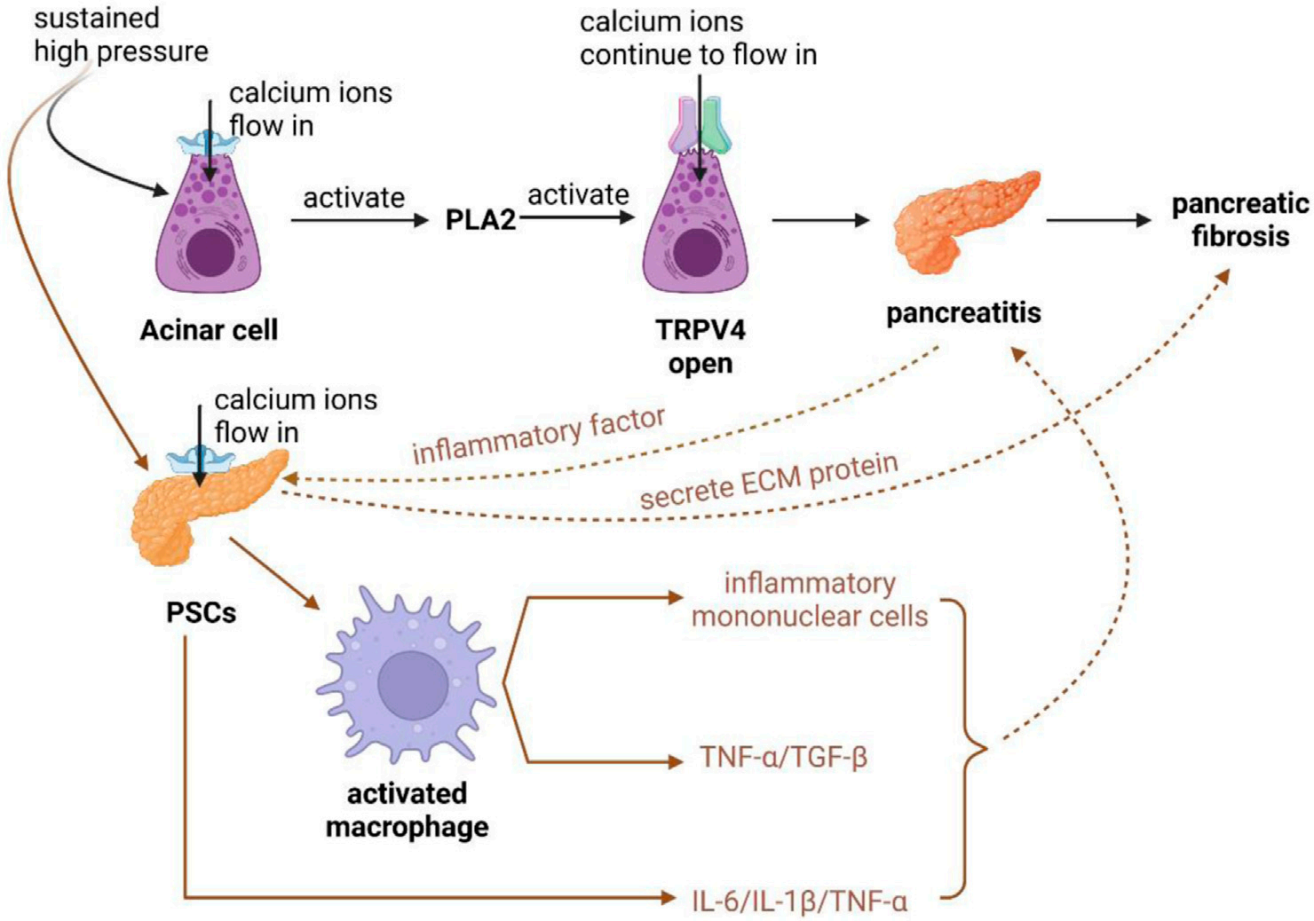
Schematic diagram of *Piezo1* channels in pancreatic alveolar cells and PSCs cells associated with pancreatic fibrosis. (1) After prolonged high pressure is applied to the alveolar cells, the opening of *Piezo1* channels activates PLA2 channels, which in turn induces the opening of TRPV4 channels, which ultimately allows for the sustained inward flow of calcium ions, causing pancreatitis with concomitant pancreatic fibrosis. (2) After the continuous action of high pressure on PSCs, PSCs were activated to secrete cellular inflammatory factors such as IL-6, IL-1β, TNF-α, etc., which could accelerate the damage of alveolar cells and lead to pancreatitis complicated by pancreatic fibrosis. Meanwhile, PSCs can activate macrophages to recruit inflammatory monocytes, and secrete TNF-α and TGF-β, which also accelerate the development of pancreatic fibrosis by promoting inflammation. (3) PSCs can secrete ECM proteins leading to pancreatic fibrosis, and after pancreatitis occurs, pancreatic alveolar cells can activate PSCs by secreting cellular inflammatory factors, accelerating the development of pancreatic fibrosis.

When patients suffer from chronic pancreatitis, it is usually associated with pancreatic fibrosis (Shimizu, 2008; Swain et al., 2022). First, after high-pressure acts on pancreatic alveolar cells, *Piezo1* channels open and calcium ions flow inward into the cells, but some experiments have found that the opening of *Piezo1* channels can only trigger transient calcium ion inward flow, which is not enough to cause pancreatitis, so only after prolonged high pressure acts on alveolar cells, *Piezo1* channels open, inducing *PLA2* channel activation, and then inducing *TRPV4* channel opening, which eventually allows a continuous inward flow of calcium ions (Romac et al., 2018; Swain et al., 2020; Gorelick and Nathanson, 2020). The high intracellular concentration of calcium ions activates trypsin and disrupts zymogen granules, leading to damage of the alveolar cells and pancreatitis, complicated by pancreatic fibrosis (Figure 3) (Hu et al., 2016).

Pancreatic fibrosis is caused by ECM deposition proteins produced by PSCs, and at the same time, PSCs can secrete pro-inflammatory cytokines to aggravate pancreatitis complicated by fibrosis. PSCs express *Piezo1*, which is activated by the continuous action of high pressure on PSCs, secreting interleukin-6 (IL-6), interleukin-1β (IL-1β), tumor necrosis factor-α (TNF-α) and other cellular inflammatory factors, which can accelerate the damage of alveolar cells and lead to pancreatitis complicated by pancreatic fibrosis (Talukdar and Tandon, 2008; Piao et al., 2015; Hao et al., 2017). At the same time, PSCs can activate macrophages to recruit inflammatory monocytes. Meanwhile, PSCs can activate macrophages to recruit inflammatory monocytes (a regulator of fibrosis) and secrete tumor necrosis factor-α (TNF-α) and transforming growth factor-β (TGF-β), which likewise accelerate pancreatic fibrosis by promoting the onset of inflammation. After the onset of pancreatitis, pancreatic follicular cells can activate PSCs by secreting cytosolic inflammatory factors to accelerate the development of pancreatic fibrosis (Figure 3) (Kuntze et al., 2020; Swain et al., 2022).

In the pancreas, *Piezo1* activation is triggered by external mechanical damage, leading to an abnormal increase in intracellular calcium ions and ultimately triggering pancreatic fibrosis. Thus, upregulation of *Piezo1* precedes the onset of fibrosis. After pancreatic cells are mechanically damaged, the *Piezo1* channel will continue to open, causing an increase in intracellular calcium ions, triggering the PLA2 pathway, and ultimately leading to the opening of the TRPV4 pathway, increasing intracellular calcium ion concentration, inducing cell self-digestion and pancreatic cell damage. Furthermore, macrophages exacerbate the development of fibrosis and pancreatitis through the production of inflammatory factors.

### 3.4 *Piezo1* and cardiac fibrosis

When the heart is diseased, it is often accompanied by cardiac fibrosis (Frangogiannis, 2021; Bartoli et al., 2022), like heart failure (Liu M. et al., 2021; Oppedisano et al., 2021), myocardial infarction (Ma et al., 2021; Zaidi et al., 2021), and hypertension (Pinho, 2019; Siamwala et al., 2020). The key characteristic of cardiac fibrosis is ECM deposition. (Ma et al., 2018; Maruyama and Imanaka-Yoshida, 2022; Sarohi et al., 2022). Cardiac fibroblasts play a crucial part in the synthesis and metabolism of ECM. These fibroblasts secrete collagen proteins to form ECM. When pathological conditions persist, excessive ECM synthesis is induced by fibroblasts, leading to ECM deposition and subsequent cardiac fibrosis. This impairs cardiac compliance and diastolic function (Frangogiannis, 2021; Liu M. et al., 2021; Kurose, 2021; Shao et al., 2022). Additionally, under pathological conditions, fibroblasts can proliferate and differentiate into myofibroblasts (MFs), and prolonged injury can also contribute to the occurrence of cardiac fibrosis (Nagpal et al., 2016; Frangogiannis, 2019; Tarbit et al., 2019).

Studies indicated that *Piezo1* is widely distributed in cardiac tissues and plays a crucial part in cardiac fibrosis. *Piezo1* is expressed in cardiac fibroblasts (CF) (Stewart and Turner, 2021), and its dysregulation, either overexpression or silencing, can lead to calcium ion defects and ROS signaling dysregulation (Ma et al., 2013; Zhu et al., 2020; Jiang F. et al., 2021; Yan et al., 2022). Mechanical stimulation that activates *Piezo1* channels can trigger calcium-mediated activation of calpains and calcineurin (Garcia-Dorado et al., 2012), leading to fibroblast-to-myofibroblast transition (Beech and Kalli, 2019; Xing et al., 2023).

**FIGURE 4:**
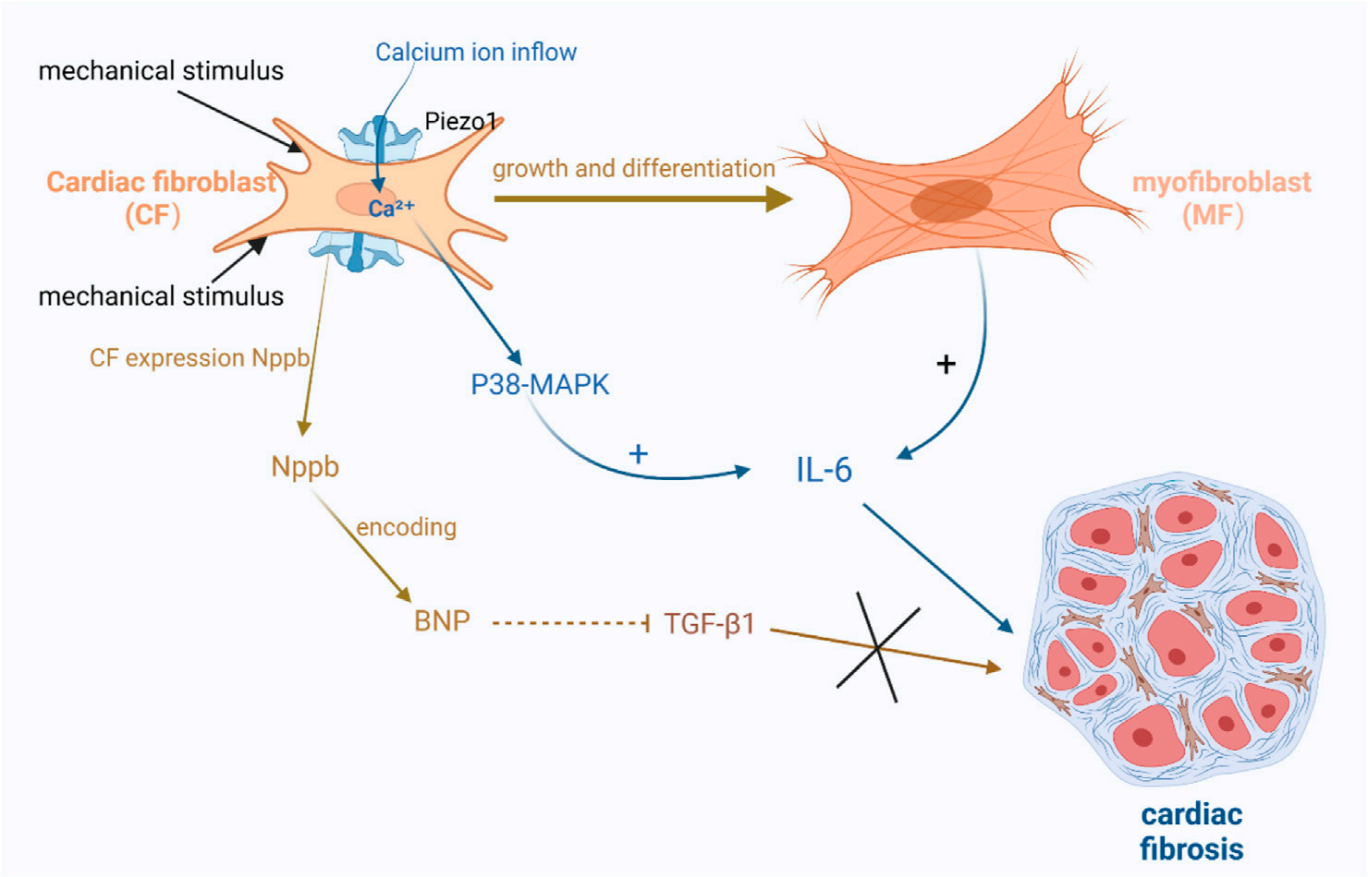
Schematic diagram of the mechanism by which *Piezo1* channels in cardiac fibroblasts are associated with cardiac fibrosis. (1) Activation of *Piezo1* triggers the activation of calcium ions and promotes fibroblasts into myofibroblasts, which are capable of secreting cytokines, including IL-6, etc. Increased calcium ions activate the downstream signaling pathway p38-MAPK, thereby increasing the level of IL-6. (2) After mechanical stimulation of *Piezo1* channel opening, *Nppb* responded to mechanical stretching by expressing BNP, which inhibited TGF-β1 and also further inhibited the promotion of cardiac fibrosis by TGF-β1.

Some studies have suggested a close relationship between *Piezo1* and interleukin-6 (IL-6), which is a pro-fibrotic cytokine (Blythe et al., 2019). Thus, the activation of *Piezo1* may induce fibroblast fibrosis through paracrine signaling involving IL-6 (Blythe et al., 2019; Emig et al., 2021; Malko et al., 2023). Experimental evidence has shown that *Piezo1* activation can trigger calcium ion activation and promote fibroblast proliferation and differentiation into myofibroblasts, which are capable of secreting cytokines, including IL-6 (Bartoli et al., 2022; Braidotti et al., 2022a). Moreover, researchers have also found that *Piezo1* activation results in increased intracellular calcium levels, subsequently activating downstream signaling pathways like p38- MAPK, resulting in elevated IL-6 levels (Figure 4) (Blythe et al., 2019; Bartoli et al., 2022; Braidotti et al., 2022a).

There is also evidence suggesting the involvement of brain natriuretic peptide (BNP) in cardiac fibrosis. BNP is abundantly present in cardiomyocytes and is known to inhibit collagen production and fibroblast proliferation (Hall, 2004; Goetze et al., 2020; Sun et al., 2023). Recent studies have identified BNP expression in fibroblasts. Fibroblasts express *Nppb*, which responds to mechanical stretch (Tsuruda et al., 2002; Ploeg et al., 2021). *Nppb* is the gene encoding BNP. Animal models have shown that the activation of Piezo1 using Yoda1, an agonist, increases *Nppb* and *Tgf-β1*. Conversely, silencing *Piezo1* expression suppresses the expression of these two genes, indicating that *Piezo1* mediates *Nppb* and *Tgf-β1* in cardiac fibroblasts under mechanical stretch stimulation. *Piezo1*, as a mechanosensitive channel, has a crucial function in regulating the mechanical stress response in cardiac fibroblasts (Bartoli et al., 2022; Braidotti et al., 2022a). Upon mechanical stimulation and opening of the *Piezo1* channel, *Nppb* reacts to it and expresses BNP, which inhibits TGF-β1 as well as further suppresses *Acta2* induction by TGF-β1. *Tgf-β1* is a gene involved in fibrosis and inflammation (Figure 4) (Paulus and Tschöpe, 2013; Tian et al., 2019).

*Piezo1* is widely distributed in cardiac tissue, and its dysregulation can lead to calcium ion defects and dysregulation of ROS signaling. Mechanical stimulation activates *Piezo1* channels, triggering calcium-mediated activation of calpain and calcineurin, leading to the transformation of fibroblasts into myofibroblasts. *Piezo1* is expressed in cardiac fibroblasts (CF), and activation of Piezo1 can trigger the transformation of fibroblasts into myofibroblasts, a key step in fibrosis. Therefore, the upregulation of *Piezo1* plays a role in the fibrosis process. Furthermore, activation of *Piezo1* may induce fibroblast fibrosis through paracrine signaling involving IL-6.

In summary, in most cases, *Piezo1* activation is triggered by mechanical stimulation, both occur through calcium influx but induce fibrosis through different signaling pathways. In pulmonary, renal, and cardiac fibrosis, upregulation of *Piezo1* may occur during the fibrotic process, whereas in pancreatic fibrosis, activation of *Piezo1* is triggered by external mechanical injury and may occur before the fibrotic process. Notably, these processes may differ in different disease states and time points.

## 4 *Piezo1* as a prospective treatment target for fibrotic diseases

*Piezo1* attracts widespread attention as a potential target for fibrotic diseases. Fibrosis is a pathological condition involving excessive ECM deposition and abnormal remodeling of tissue structure. Several studies have attempted to inhibit the fibrotic process by inhibiting the activity of *Piezo1* channels. The development of fibrosis is attenuated by interfering with *Piezo1* channel function or blocking *Piezo1* channel-related signal pathways, like the calcium pathway and intracellular signal pathways, through the use of specific *Piezo1* channel antagonists or inhibitors. In addition to inhibiting *Piezo1* channel activity, studies have also been conducted to enhance the function of *Piezo1* channels through the use of agonists or promoters or to adjust the activity level of *Piezo1* channels by the use of modulators, to achieve regulation of the fibrotic process. In addition to directly targeting *Piezo1* channels, several studies are exploring other therapeutic strategies related to *Piezo1*. For example, researchers continue to identify downstream signaling pathways and molecular targets that can influence *Piezo1* regulation and are banking on controlling downstream pathways and signals to achieve intervention in the fibrotic process.

### 4.1 *Piezo1* as a prospective treatment target for pulmonary fibrosis disease

Recent studies have highlighted the great importance of Piezo1 channels in the EMT process, suggesting that they may serve as key components mediating TGF-β signaling and epithelial cell transformation. This not only contributes to a deeper understanding of EMT-related diseases such as pulmonary fibrosis but may also provide new targets for the development of therapeutic strategies (Huang et al., 2021a; Zhang Y. A. et al., 2021). Besides, Mechanical ventilation is extensively used in critically ill patients, but at the same time, it may trigger and exacerbate the progression of pulmonary fibrosis. It was discovered that *Piezo1* channels are activated by mechanical stretch under conditions of mechanical ventilation, leading to a cascade of cellular signaling events. This process is mediated through the activation of the RhoA/ROCK1 signaling pathway, which in turn triggers an increase in intracellular calcium ion concentration and leads to Bcl-2 inhibition, which in turn induces apoptosis in type II lung cells (Liang et al., 2019; Zhang Y. A. et al., 2021; Jiang et al., 2021b). Mechanical stretch activation of *Piezo1* induces type II lung cell apoptosis via Ca2+ inward flow (Zhang et al., 2021c). In ARDS, Piezo1 and Ca2+ inward flow are thought to have a potential role (Liang et al., 2019; Jiang et al., 2021c; Fang et al., 2022). Future in-depth studies are expected to reveal the fine mechanisms of these pathways and provide more insight into the development of therapeutic strategies.

In summary, the essential role of the Piezo1 pathway in lung diseases should not be overlooked, and further studies on its molecular mechanism will provide a basis for drug development and optimization of therapeutic approaches. This promising research direction is expected to bring new hope for the future development of lung disease treatment.

### 4.2 *Piezo1* as a prospective treatment target for renal fibrosis disease

For the potential link between *Piezo1* and renal fibrosis, several studies have provided evidence suggesting that *Piezo1* can be activated by mechanical stretch, chemical stimuli, or increased synthesis of extracellular matrix (ECM). Additionally, inhibition of *Piezo1* expression in animals has been demonstrated to alleviate fibrotic processes in the kidney, this provides preliminary evidence for the feasibility of *Piezo1* as a prospective treatment for renal fibrosis (Zhao X. et al., 2022). These findings, along with the previously described signaling pathways associated with *Piezo1* and renal fibrosis, strongly suggest that *Piezo1* plays a significant part in renal fibrosis. Collagen deposition or cross-linking leads to increased ECM stiffness and accelerated ECM secretion, which in turn aggravates the renal fibrosis process, forming a vicious positive feedback loop. A potential therapeutic strategy has been proposed to target ECM stiffness-induced mechanotransduction signaling pathways. By interfering with the mechanotransduction signaling pathway, it is expected to inhibit the increase in ECM stiffness, thereby slowing down or reversing the process of renal fibrosis (Seghers et al., 2016; Fu et al., 2021; He et al., 2022a; Zhao X. et al., 2022). Although this therapeutic strategy still needs further research and validation, it provides a new direction and idea for the treatment of renal fibrosis.

### 4.3 *Piezo1* as a prospective treatment target for pancreatic fibrosis disease

Elevated pancreatic duct pressure leads to fibrosis mediated by *Piezo1*-activated PSCs. In a mouse model, the action of *Piezo1* activator Yoda1 on PSCs leads to increased fibrosis, while the action of *Piezo1* inhibitor GsMTx4 attenuates the fibrotic response. It can be speculated that the blocker of *Piezo1* is used to act on PSCs as a target to attenuate pancreatic fibrosis (Kuntze et al., 2020; Swain et al., 2022). In addition to this, it has been suggested that Piezo1 stimulates PLA2, which initiates the TRPV4 pathway, and we can also use the blocker of TRPV4 to attenuate the damage to pancreatic cells (Swain et al., 2020). Further studies will contribute to a better discovery of the mechanism of *Piezo1* in pancreatic fibrosis and develop new therapeutic options (Zhan and Li, 2018; Swain et al., 2020; Gorelick and Nathanson, 2020; Swain et al., 2022).

### 4.4 *Piezo1* as a prospective treatment target for cardiac fibrosis disease

*Piezo1* takes a mechanosensing part in cardiac fibroblasts, and we suggest that *Piezo1* may be a prospective target to attenuate fibrosis in abnormal pathological states of the heart and maybe a potential target to interfere with cardiac fibroblast function (Zhang et al., 2021a; Jiang F. et al., 2021; Braidotti et al., 2022a). *Piezo1* has a crucial function in cardiac fibrosis and provides an idea for the attenuation, cessation, or prevention of cardiac fibrosis. On the one hand, we can start from the perspective that after the mechanical activation of *Piezo1*, the *Nppb* gene in fibroblasts expresses BNP to anti-fibroblasts, and through the anti-fibroblast effect of BNP, we can attenuate or even prevent the occurrence of fibroblasts ahead of time (Ploeg et al., 2021), and on the other hand, we can also start from the calcium inward flow triggered by *Piezo1* and the P38-MAPK signaling pathway, which affects the release of cytokines related with fibroblasts formation, to modulate fibroblasts occurrence (Blythe et al., 2019; Emig et al., 2021). In addition, we have compiled a Table 1 detailing the pro fibrotic and antifibrotic effects of peizo1 in the context of renal, pancreatic, cardiac and pulmonary fibrosis.

**TABLE 1:** Pro-or anti-fibrotic effects of Piezo1 in the context of pulmonary, renal, pancreatic, and cardiac fibrosis.

Disease	Action factor or pathway	Pro- or anti-fibrotic	Ref.
Pulmonary fibrosis	TGF-β-MAPK/smad	Pro-fibrotic	Huang et al. (2021c)
Renal fibrosis	Piezo1-P38-MAPK-YAP	Pro-fibrotic	Fu et al. (2021)
Pancreatic fibrosis	Piezo1-PLA2-TRPV4	Pro-fibrotic	Swain et al. (2020); Gorelick and Nathanson (2020)
	IL-6, IL-1β, TNF-α, etc	Pro-fibrotic	Talukdar and Tandon (2008); Piao et al. (2015); Hao et al. (2017)
Cardiac fibrosis	IL-6	Pro-fibrotic	Blythe et al. (2019); Emig et al. (2021); Malko et al. (2023)
	BNP	Anti-fibrotic	Goetze et al. (2020); Sun et al. (2023)

In conclusion, *Piezo1* could be a potential target for pulmonary fibrosis, renal fibrosis, pancreatic fibrosis, and cardiac fibrosis. The difficulty associated with treating fibrotic diseases often lies in reversing them, and we are aware of the seriousness of persistent fibrosis in the heart, lungs, liver, and kidneys. Although *Piezo1* provides us with a novel direction for treating fibrotic diseases, its current research and application are mostly limited to animal models. Considering the differences between humans and animals, it will take a long time to obtain effective results from *Piezo1* for the treatment of fibrotic diseases, and we expect Piezo1 to bring hope to fibrotic patients sooner.

## 5 Summary and discussion

Fibrosis is a clinically advanced presentation of the majority of diseases and is a common phenomenon after organ damage with failure, severely affecting the wellbeing of patients. Therefore, using effective methods to inhibit or slow down the progression of disease fibrosis has attracted extensive attention from researchers. Due to the complex pathological mechanisms of fibrosis, it is crucial to further explore reliable therapeutic approaches. *Piezo1*, a key molecule in fibrosis, has been shown to exert an essential role in many types of fibrotic diseases. Hence, we expect that future studies should be devoted to further elucidating the specific mechanisms of Piezo1’s role in different fibrotic diseases, as well as its inter-regulatory relationship with other crucial signaling pathways. On this basis, the development of specific antagonists targeting *Piezo1* will be a potential therapeutic strategy to provide new ideas for the clinical treatment of fibrotic diseases and open up new possibilities for the treatment of fibrotic diseases.

## Abbreviations

MA, Mechanically-Activated; ECM, Extracellular matrix; TGF-β, Transforming Growth Factor-beta; MAPK, Mitogen-Activated Protein Kinase; TM, Transmembrane; CTD, C-terminal structural domain; CED, C-terminal extracellular structural domain; PF, Pulmonary fibrosis; EMT, Epithelial-mesenchymal transition; ARDS, Acute respiratory distress syndrome; MV, Mechanical stretch; ATI, Alveolar epithelial type I; ATII, Alveolar epithelial type II; AJs, Adhesion junctions; UUO, Unilateral ureteral obstruction; HK2 cells, Human renal cortical proximal tubular epithelial cells; mptc, Mouse proximal tubule cells; α-SMA, α-Smooth Muscle Actin; YAP, Yes-associated protein; PSCs, Pancreatic stellate cells; PLA2, Phospholipase A2; TRPV4, Transient receptor potential vanilloid 4; TNF-α, tumor necrosis factorα; IL-6, Interleukin-6; IL-1β, Interleukin-1β; MFs, Myofibroblasts; CF, Cardiac fibroblasts; BNP, Brain natriuretic peptide; ROS, Reactive Oxygen Species.
