# Corrigendum: Mechanotransductive receptor *Piezo1* as a promising target in the treatment of fibrosis diseases

**DOI:** 10.3389/fmolb.2024.1420585

**Published:** 2024-05-16

**Authors:** Yi Xu, Yiqian Huang, Xiaoqing Cheng, Bin Hu, Danling Jiang, Lidong Wu, Shengliang Peng, Jialing Hu

**Affiliations:** ^1^ Department of Emergency Medicine, The Second Affiliated Hospital of Nanchang University, Nanchang, China; ^2^ The Second Affiliated Hospital of Nanchang University, The Second Clinical Medical College of Nanchang University, Nanchang, China; ^3^ Department of Ultrasound Medicine, The Second Affiliated Hospital of Nanchang University, Nanchang, China; ^4^ Department of Anesthesiology, The Second Affiliated Hospital of Nanchang University, Nanchang, China

**Keywords:** *Piezo1*, *Piezo2*, therapeutic target, fibrosis, Ca^2+^

In the published article, there were two errors in the **References**.

The reference for “**Zhao et al., 2022b**” was incorrectly written as “Zhao, M., Wang, L., Wang, M., Zhou, S., Lu, Y., Cui, H., et al. (2022b). Targeting fibrosis, mechanisms and clinical trials. *Signal Transduct. Target Ther.* 7 (1), 206. doi:10.1038/s41392-022-01070-3”. It should be “Zhao, X., Kong, Y., Liang, B., Xu, J., Lin, Y., Zhou, N., et al. (2022) Mechanosensitive Piezo1 channels mediate renal fibrosis. *JCI Insight*. 7 (7), e152330. doi: 10.1172/jci.insight.152330”. The respective in-text citation has also been corrected to “Zhao X. et al., 2022”.

The reference for “**Soattin et al., 2014**” was incorrectly written as “Soattin, L., Ling, J., Jia, Z., Coyle, D., and Gu, J. G. (2014). Merkel cells transduce and encode tactile stimuli to drive Aβ-afferent impulses. *Cell* 157 (3), 664–675. doi:10.1016/j.cell.2014.02.026”. It should be “Soattin, L., Fiore, M., Gavazzo, P., Viti, F., Facci, P., Raiteri, R., et al. (2016) The biophysics of piezo1 and piezo2 mechanosensitive channels. *Biophys Chem.* 208, 26-33. doi: 10.1016/j.bpc.2015.06.013”.

The authors apologize for these errors and state that these do not change the scientific conclusions of the article in any way. The original article has been updated.

